# Induction of cytokines in different organs after intranasal inoculation of *Campylobacter jejuni* in mice

**DOI:** 10.1186/1757-4749-4-23

**Published:** 2012-12-17

**Authors:** Nadia A Al-Banna, Raj Raghupathy, M John Albert

**Affiliations:** 1 Department of Microbiology, Faculty of Medicine, Kuwait University, PO Box 24923, Safat 13110, Kuwait

**Keywords:** *Campylobacter jejuni*, Cytokines, Systemic, Mouse lung model

## Abstract

**Background:**

Cytokine production and histopathological changes occur in the lungs of mice after intranasal inoculation with *Campylobacter jejuni*, but the levels of cytokines in different organs to which *C. jejuni* disseminates have not been studied.

**Findings:**

Adult BALB/c mice were intranasally inoculated with *C. jejuni* 81*–*176 (test) or phosphate-buffered saline (control) (n=16 per group). The levels of cytokines in the organs (spleen, liver, and small and large intestines) to which *C. jejuni* disseminated were measured by ELISA. Two cytokine patterns were observed. First, increased proinflammatory cytokines, TNF-α, IL-1, and IL-2, were followed by anti-inflammatory cytokines, IL-4 and IL-10 in the spleen and large intestine. Second, in the liver and small intestine, there was a predominant production of anti-inflammatory cytokines, IL-4 and IL-10, with some increase in IL-2 levels. In the spleen and intestines, the levels of pro- and anti-inflammatory cytokines were concurrently increased.

**Conclusion:**

Dissemination of *C. jejuni* is associated with the production of different cytokine profiles in different tissues, with the proinflammatory response appearing in the spleen and large intestine at an earlier time point than in the liver and small intestine. The organs produce different cytokine profiles in response to *C. jejuni* dissemination. These preliminary findings should be confirmed with a study involving a larger group of animals.

## Findings

### Background

The extraintestinal manifestations of *Campylobacter jejuni*, an enteropathogenic bacterium [1], are reported in some patients [2]. *C. jejuni* invades epithelial cells in vitro [3], and can be isolated from the spleen and liver in infected animals [4,5]. In vitro studies demonstrated the production of proinflammatory cytokines in *C. jejuni*-infected monocytic [6], dendritic [7] and intestinal epithelial cell lines [8], peripheral blood mononuclear cells [9] and splenocytes [10]. The association between cytokine production and disease protection/resolution, suggested in patients [9], was shown by the increased susceptibility of mice deficient in MyD88 or NF-κB to *C. jejuni*[11,12]. However, cytokine responses after *C. jejuni* dissemination were not characterised in vivo.

In the current study, we used the mouse lung model to examine whether different tissues produce cytokines after *C. jejuni* disseminates from the primary site of infection. This model was used to study the pathogenesis and immunity of *C. jejuni*[13-15] and other enteric pathogens [16,17]. Also, we previously studied the histopathological changes and the cytokines produced in the lungs after *C. jejuni* infection [18], and *C. jejuni* dissemination to several organs [19]. Though the lung model is an unnatural model for enteric pathogens, it is a relevant model, as the lungs and the gut are part of a common mucosal system and oral infection induces immunity that protects other mucosal sites including the lungs [20].

## Materials and methods

A lung model of *C. jejuni* infection was used in adult mice [18,19]. The experiments were conducted according to institutional guidelines after ethical committee approval. Briefly, adult BALB/c mice were intranasally inoculated with ~4 × 10^9^ cfu (colony forming units) of *C. jejuni* strain 81–176 (n=16, test) or phosphate-buffered saline (PBS, pH 7.2) (n=16, control). After 1–10 days, blood and several organs (spleen, liver, and small and large intestines) of mice (from 4 mice each from test and control groups sacrificed at each time point) were collected, weighed and placed on ice. The organs were homogenised in PBS using a pestle and mortar under sterile condition in a biosafety cabinet. Homogenates were resuspended in 1% Triton X-100 (Sigma, St. Louis, MO, USA) on ice and centrifuged at 4°C at 19,319 × *g* for 10 min in a Beckman J2-MI centrifuge using a JA 20.1 rotor (Beckman, Fullerton, CA, USA). Sera and supernatants were stored at −80°C.

Cytokines (TNF-α, IL-1, IL-2, IL-4, and IL-10) were assessed in duplicate samples of sera and tissue homogenates using ELISA, according to manufacturer’s instructions. Kits were obtained from Endogen (Pierce Biotechnology, Rockford, IL, USA) and Biosource (Biosource International, Invitrogen, Camarillo, CA, USA). Absorbance values were read at 450 λ using an ELISA reader (Labsystems Multiscan MS, Finland). Cytokine levels were expressed in pg/ml of serum, or pg/100 mg of tissue: (pg/ml × volume of homogenate in ml)/(weight in grams × 10). As the data obtained were not normally distributed, Mann–Whitney *U* test was used for non-parametric comparison, and the difference was considered significant with *P* value ≤ 0.05.

The cytokine production in the lungs and the histopathology of the lungs of these animals have been previously reported [18,19].

## Results

### *C. jejuni* induces proinflammatory cytokines in the spleen and large intestine at an earlier time point than in the liver and small intestine

*C. jejuni* was isolated from the spleen, liver and serum on day 1, and from the small and large intestines for up to day 5 post-intranasal inoculation [19]. We observed the production of TNF-α, IL-1 and IL-2 in these organs at different time points (Table 1). Initially, the levels of proinflammatory cytokines were increased in the spleen, large intestine and serum. On day 1, TNF-α levels were increased by 4–12 fold in all organs, IL-2 levels were increased in the spleen and IL-1 levels were increased in the sera of test animals compared to those in control animals (P<0.05). On day 3, the IL-1 level was increased by 2.5-fold in the spleen, and on day 5, the levels of IL-1 and/or IL-2 were significantly increased in the spleen, large intestine and serum of test mice (P<0.05). However, in the liver and small intestine, the levels of IL-1 and/or IL-2 were increased only on day 3 (P<0.05), and the level of TNF-α was similar to that in controls.

**Table 1:** **Levels of proinflammatory cytokines, TNF-α, IL-1 and IL-2, in intranasally infected mice with *C. jejuni *(mean ± SD)**

**Organ**	**Group**	**Day 1**			**Day 3**			**Day 5**			**Day 10**		
		**TNF-α**	**IL-1**	**IL-2**	**TNF-α**	**IL-1**	**IL-2**	**TNF-α**	**IL-1**	**IL-2**	**TNF-α**	**IL-1**	**IL-2**
Spleen	Test	1042 (±32) *	270 (±59)	153 (±39)*	478 (±340)	861 (±387)*	181 (±16)	114 (±16)	1094 (±208) *	70 (±39)	138 (±26)	852 (±183)	78 (±19)
	Control	245 (±31)	814 (±262)	87 (±27)	1045 (±57)	319 (±89)	105 (±51)	218 (±65)	717 (±62)	170 (±92)	182 (±16)	1186 (±381)	55 (±13)
Large intestine	Test	110 (±59)*	111 (±12)	471 (±655)	20 (±5)	147 (±127)	1634 (±918)	13 (±3)	161 (±40)*	5752 (±1251)*	6 (±7)	101 (±35)	ND
	Control	13 (±6)	141 (±10)	5416 (±1057)	109 (±61)	144 (±59)	1707 (±739)	15 (±11)	62 (±47)	2216 (±705)	16 (±1)	159 (±44)	ND
Liver	Test	186 (±17)	50 (±21)	256 (±129)	4581 (±4244)	133 (±33)*	1209 (±745)*	3860 (±1000)	118 (±34)	389 (±69)	ND	126 (±7)	64 (±22)
	Control	4567 (±1992)	73 (±25)	718 (±297)	4425 (±992)	79 (±26)	458 (±116)	4183 (±273)	142 (±72)	366 (±102)	ND	148 (±31)	55 (±8)
Small intestine	Test	7 (±6)	12 (±9)	2483 (±286)	11 (±2)	6 (±0)	2063 (±428)*	6 (±2)	12 (±2)	5427 (±756)	13 (±3)	4 (±1)	3640 (±1737)
	Control	16 (±13)	142 (±37)	7352 (±1909)	11 (±2)	11 (±6)	830 (±335)	11 (±1)	112 (±25)	5012 (±828)	10 (±5)	2 (±1)	6603 (±379)
Serum	Test	101 (±9)*	55 (±13)*	8 (±0)	28 (±39)	27 (±2)	8 (±0)	76 (±54)	23 (±4)*	8 (±0)	45 (±39)	23 (±1)	8 (±0)
	Control	29 (±14)	8 (±9)	8 (±0)	100 (±5)	16 (±11)	8 (±0)	43 (±36)	3 (±0)	8 (±0)	85 (±14)	28 (±3)	8 (±0)

* Indicates significant difference (*P≤* 0.05) in values between test and control mice; ND, not done.

### *C. jejuni* induces anti-inflammatory cytokines in all organs by day 5

Anti-inflammatory cytokines were most apparent on day 5, with a 2-fold increase in the IL-4 levels in the spleen, liver, large intestine and serum, and a 2–20 fold increase in the IL-10 levels in the liver, and small and large intestines (Table 2). There was an early rise in the levels of anti-inflammatory cytokines in the spleen and serum, and small intestine on days 1, and 3, respectively.

**Table 2:** **Levels of anti-inflammatory cytokines, IL-4 and IL-10, in intranasally infected mice with *C. jejuni *(mean±SD)**

**Organ**	**Group**	**Day 1**		**Day 3**		**Day 5**		**Day 10**	
		**IL-4**	**IL-10**	**IL-4**	**IL-10**	**IL-4**	**IL-10**	**IL-4**	**IL-10**
Spleen	Test	15277 (±6630)	314 (±32) *	22824 (±3489)	326 (±125)	40284 (±10363)*	364 (±38)	9509 (±3368)	190 (±129)
	Control	23230 (±3829)	239 (±34)	16706 (±7430)	354 (±54)	21164 (±1355)	433 (±201)	18770 (±4750)	130 (±49)
Large intestine	Test	1 (±1)	2 (±0)	4 (±3)	10 (±11)	31 (±31)*	103 (±113)*	5 (±1)	12 (±3)
	Control	3 (±1)	13 (±10)	6 (±5)	37 (±6)	6 (±3)	13 (±12)	8 (±2)	6 (±4)
Liver	Test	4960 (±5721)	90 (±14)	15856 (±15787)	122 (±21)	13820 (±3241)*	276 (±45)*	ND	216 (±14)
	Control	20819 (±6306)	148 (±23)	5033 (±3280)	147 (±40)	6380 (±2833)	138 (±45)	ND	211 (±68)
Small intestine	Test	15 (±10)	2 (±0)	31 (±4)*	7 (±7)	19 (±10)	23 (±15)*	11 (±3)	11 (±9)
	Control	23 (±11)	2 (±0)	15 (±9)	8 (±5)	23 (±11)	2 (±1)	22 (±5)	6 (±4)
Serum	Test	786 (±194)*	339 (±70)	239 (±85)	152 (±118)	435 (±70)*	48 (±31)	335 (±108)	113 (±70)
	Control	323 (±29)	236 (±118)	1690 (±123)	484 (±321)	284 (±78)	304 (±157)	409 (±123)	323 (±128)

* Indicates significant difference (*P≤* 0.05) in values between test and control mice; ND, not done.

## Discussion

This is the first study to report cytokine responses induced in different organs over an extended period after *C. jejuni* dissemination (summarised in Table 3). The proinflammatory cytokine response was induced in the spleen and large intestine before the response in the liver and small intestine. For example, the level of TNF-α was increased in the spleen, large intestine and serum, but not in the liver and small intestine on day 1, although the organism was isolated from these tissues [19]. IL-1 levels were increased in the spleen, large intestine and serum on day 3 and/or 5, but only on day 3 in the liver, and remained unchanged in the small intestine.

**Table 3:** **Summary of observed cytokine profiles and bacterial dissemination in intranasally infected mice *with C. jejuni***

**Organ**	**Observation**	**Day 1**	**Day 3**	**Day 5**	**Day 10**
Spleen	Proinflammatory cytokine	TNF-α, IL-2	IL-1	IL-1	-
	Anti-inflammatory cytokine	IL-10	-	-	-
	*C. jejuni* isolated	Yes	No	No	ND
Large intestine	Proinflammatory cytokine	TNF-α	-	IL-1, IL-2	-
	Anti-inflammatory cytokine	-	-	IL-4, IL-10	-
	*C. jejuni* isolated	Yes	Yes	Yes	ND
Liver	Proinflammatory cytokine	-	IL-1, IL-2	-	-
	Anti-inflammatory cytokine	-	-	IL-4, IL-10	-
	*C. jejuni* isolated	Yes	No	No	ND
Small intestine	Proinflammatory cytokine	-	IL-2	-	-
	Anti-inflammatory cytokine	-	IL-4	IL-10	-
	*C. jejuni* isolated	Yes	Yes	Yes	ND
Serum	Proinflammatory cytokine	TNF-α, IL-1	-	IL-1	-
	Anti-inflammatory cytokine	IL-4	-	IL-4	-
	*C. jejuni* isolated	Yes	No	No	ND

Mention of cytokine indicates that cytokine level in test mice was significantly increased compared to in control mice; ND, culture not done;-, no difference between test mice and control mice.

We detected cytokines in the serum after intranasal inoculation, as others have found in intraperitoneal or oral models [21,22]. However, the cytokine profile in the serum was not representative of organs. Indeed, the serum IL-1 level on day 1 did not correspond to any organ, and serum levels on day 5 coincided with those in the spleen and large intestine only.

Before cytokine normalisation on day 10, the levels of IL-4 and IL-10 were increased by day 5. This early increase is difficult to interpret, especially when it coincided with increased proinflammatory cytokine levels, as in the spleen (day 1) or small intestine (day 3). Concurrent pro-and anti-inflammatory cytokine production was noted in an invitro model [8].

Our observations illustrate *C. jejuni*-induced cytokine responses in different organs, and may reflect cellular responses. The increased TNF-α and IL-1 levels may represent the activation of antigen presenting cells (activated macrophages and dendritic cells), and intestinal epithelial cells in response to *C. jejuni*, as in in vitro models [6-8,23]. Similarly, the increased IL-2 levels, which control reinfection of *C. jejuni* in mice [24], may reflect the activation of IL-2 responsive T cells and/or natural killer cells [25]. Examining the histopathological changes and the influx of cytokine producing cells will further our understanding of this response*.* In future, a large sample size of animals should be used to avoid the inconsistencies we observed in the levels of some cytokines, for example, reduced levels of IL-1 in the small intestine. Other cytokines (IFN-γ, IL-6, IL-17 and IL-22) and other strains of *C. jejuni* and other campylobacter species should also be studied.

Surprisingly, the colonisation status did not fully correlate with the cytokine profile (Table 3). Proinflammatory cytokine profiles varied between the different organs that had *C. jejuni* on day 1, for example, the spleen and large intestine, in contrast to the liver and small intestine. The increased anti-inflammatory cytokines and the isolation of *C. jejuni* from the small and large intestines coincided, while the production of IL-1 in the spleen, liver and serum continued beyond bacterial clearance. It is unclear if the presence of *C. jejuni* in the intestine on days 3 and 5 would provide a source of virulence factors that affect the spleen.

## Conclusion

This is the first study to demonstrate the induction of cytokine responses by different organs in mice over an extended period of time after intranasal inoculation with *C. jejuni*. The cytokine profiles of these organs differed. Some organs produced cytokines even after bacterial clearance. These findings illustrate the dynamic nature of cytokine production in different organs that can respond to *C. jejuni* dissemination, possibly reflecting their immune status. Future work should examine other cytokines and determination of cellular sources of all cytokines using a larger sample size of mice.

## Competing interests

The authors declare that they have no competing interests.

## Authors’ contributions

NA carried out the experiments and wrote the manuscript. RR participated in experimental design and revised the manuscript. MJA supervised the experimental work and revised the manuscript. All authors read and approved the final manuscript.
